# Multi-scale thermal stability of a hard thermoplastic protein-based material

**DOI:** 10.1038/ncomms9313

**Published:** 2015-09-21

**Authors:** Victoria Latza, Paul A. Guerette, Dawei Ding, Shahrouz Amini, Akshita Kumar, Ingo Schmidt, Steven Keating, Neri Oxman, James C. Weaver, Peter Fratzl, Ali Miserez, Admir Masic

**Affiliations:** 1 Department of Biomaterials, Max Planck Institute of Colloids and Interfaces, Research Campus Golm, Potsdam 14424, Germany; 2 School of Materials Science and Engineering, Nanyang Technological University, Singapore 639798, Singapore; 3 Energy Research Institute at Nanyang Technological University (ERI@N), Singapore 637553, Singapore; 4 School of Biological Science, Nanyang Technological University, Singapore 639798, Singapore; 5 MIT Media Lab, Massachusetts Institute of Technology, Cambridge, Massachusetts 02139, USA; 6 Wyss Institute for Biologically Inspired Engineering, Harvard University, Cambridge, Massachusetts 02138, USA

## Abstract

Although thermoplastic materials are mostly derived from petro-chemicals, it would be highly desirable, from a sustainability perspective, to produce them instead from renewable biopolymers. Unfortunately, biopolymers exhibiting thermoplastic behaviour and which preserve their mechanical properties post processing are essentially non-existent. The robust sucker ring teeth (SRT) from squid and cuttlefish are one notable exception of thermoplastic biopolymers. Here we describe thermoplastic processing of squid SRT via hot extrusion of fibres, demonstrating the potential suitability of these materials for large-scale thermal forming. Using high-resolution *in situ* X-ray diffraction and vibrational spectroscopy, we elucidate the molecular and nanoscale features responsible for this behaviour and show that SRT consist of semi-crystalline polymers, whereby heat-resistant, nanocrystalline β-sheets embedded within an amorphous matrix are organized into a hexagonally packed nanofibrillar lattice. This study provides key insights for the molecular design of biomimetic protein- and peptide-based thermoplastic structural biopolymers with potential biomedical and 3D printing applications.

## Abstract (web summary)

Sucker ring teeth from squid and cuttlefish represent rare examples of thermoplastic biopolymers. Here, the authors demonstrate how these materials may be processed for implementation in biomedical and 3D printing applications.

## Graphical abstract

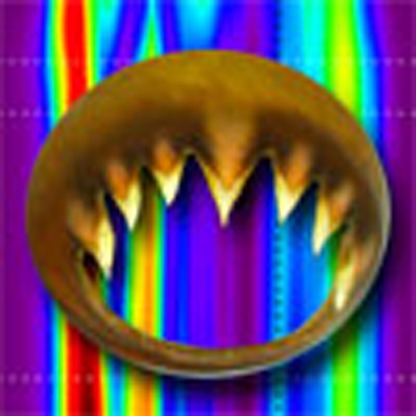

A primary reason behind the massive use of thermoplastic polymeric materials for everyday products relates to their ease of processing. Thermoplastics can be repeatedly melted and moulded into any complex shape, making them suitable for processing methods such as extrusion, injection-moulding or drawing^1^. These thermo-mechanical processing steps can be repeated multiple times over the life cycle of the material, resulting in their extensive recyclability and reusability. Unfortunately, the synthesis of thermoplastic polymers largely relies on non-renewable petrochemical products, which is a longstanding problem from a sustainability perspective^2^. Although natural biopolymers can be derived from renewable sources, their chemical processing often induces irreversible deterioration of their macroscopic physical properties and, currently, there are no common commercially available biological polymers that exhibit true thermoplastic behaviours^3^,^4^. For example, silk-based materials and devices usually involve an initial solubilization step in harsh solvents such as 9M lithium bromide or hexafluoroisopropanol^5^ and re-solubilization after initial processing is challenging. Collagen, a biopolymer that fulfills a variety of mechanical functions, exhibits limited solubility due to the high number of intermolecular cross-links^6^, and collagen extracted from animal sources such as the skin or tendons requires alkaline, acidic or enzymatic treatments that often leads to irreversible degradation. The resulting material is deficient in its characteristic hierarchical organization and integrity and is in turn much weaker than native, covalently cross-linked collagen^7^. Hybrid protein/polysaccharide biocomposites such as insect cuticles^8^ and spider fangs^9^ also exhibit high specific mechanical properties, but they are essentially insoluble in even the harshest denaturing cocktails, which is the first key step towards their processing into useful objects. Chitosan, the de-acetylated version of chitin, is conveniently soluble in weak acid conditions^10^, but its load-bearing capacity under hydrated conditions is dramatically reduced. This, however, can be partially remediated by chemically functionalizing the amine moiety, but this comes at the expense of re-processability^11^,^12^.

A notable exception are the sucker ring teeth (SRT), which line the arms and tentacles of squid and cuttlefish^13^. Each arm and tentacle contains hundreds of these ring-like structures featuring prominent tooth-like projections for use in prey capture and handling^14^. To fulfill this key functional role, SRT exhibit mechanical properties that compare with the strongest synthetic polymers, such as high-molecular-weight polyethylene^15^. In natural materials, such properties are usually obtained by load-sharing via the incorporation of a stiffer second inorganic phase such as seen in the bone or teeth^16^,^17^,^18^, or in rarer cases, by extensive covalent cross-linking between the biomacromolecular building blocks^19^,^20^. In contrast, SRT are assembled entirely from a protein family exhibiting a block co-polymer organization, with one of the block domains folding into nano-confined β-sheets of precise dimensions that carry a large fraction of the mechanical load^21^. Remarkably, inter-chain covalent cross-linking is absent and the structure is stabilized primarily through intrinsically weak hydrogen bonding. As it is stabilized by the cooperative action of weak bonds, SRT can be melted by simple heating in water^13^ and reshaped multiple times just like standard thermoplastics such as polyethylene, thus providing intriguing insights in eco-friendly processing. We first described this thermoplastic effect in 2009 using SRT isolated from the Humboldt squid, *Dosidicus gigas (D. gigas)*^15^ and these properties have more recently been verified in a related species^22^.

In the present work, we explore the molecular and physico-chemical mechanisms behind the thermoplastic behaviour of the SRT biopolymer from *D. gigas*. To tackle this question, we studied the thermo-stability of SRT with a variety of X-ray scattering, spectroscopic and nanomechanical techniques. The data show that the constituent heat-resistant nanoconfined β-sheets are thermally stable up to 220 °C and are embedded within an amorphous matrix that exhibits viscous flow at a temperature below the melting or degradation point of the crystalline phase. These results have key implications for the *de novo* design of peptide and protein-based materials that could exhibit fully thermoplastic behaviour and recyclability, which are characteristics that are directly relevant for three-dimensional (3D) bioprinting applications.

## Results

### Thermal extrusion of SRT proteins

**Figure 1:**
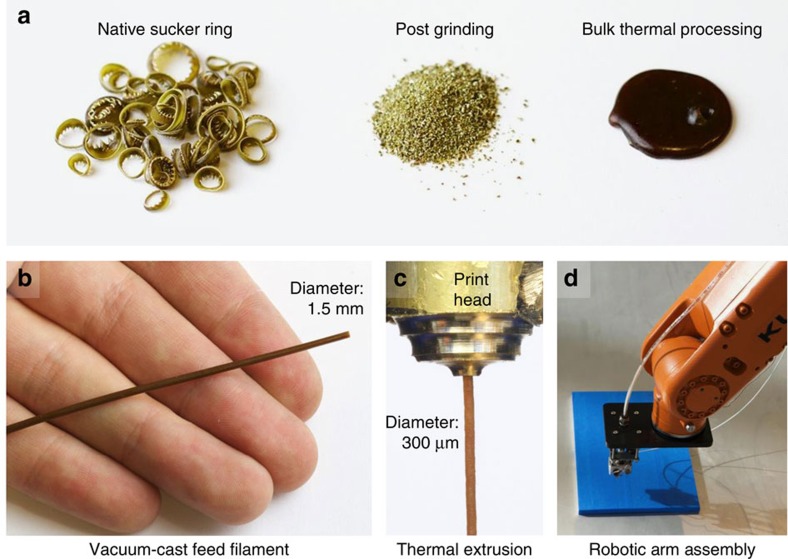
Thermal processing and filamentous extrusion of native SRT proteins. (**a**) Native SRT were ground with a mortar and pestle into a powder, mixed with plasticizing agents (50% SRT, 25% water and 25% glycerol by weight) and heated to 150 °C. The resulting molten STR mixture was then vacuum formed into a 1.5-mm diameter feed filament (**b**), and thermally extruded though a fused filament fabrication (FFF) print head attached to a robotically controlled arm to create a 300-μm diameter filament (**c**,**d**).

As an initial proof-of-concept and to demonstrate their thermoplastic behaviour, we investigated the suitability of the SRT protein mixture as a potential material for filamentous thermal extrusion in 3D printing applications. Beginning with native SRT, the material was rinsed with deionized water, dried, ground into a fine powder (below 100 μm particle size) and mixed with plasticizing agents (50% SRT, 25% water and 25% glycerol by weight). The mixture was heated to 150 °C and mechanically agitated, resulting in a viscous state suitable for moulding, casting and extrusion (Fig. 1a). The processed material was stable when cooled and could be formed into films, rods and filaments, whereas leaching and evaporation of the plasticizers resulted in increased stiffness over the following week. To enable feed filament extrusion for future 3D printing applications, a 1.5-mm-diameter filament was formed through vacuum casting in a silicone mould (Fig. 1b).

The feed filament was then extruded in a custom-made Bowden extrusion print head through brass nozzle diameters ranging from 0.3 to 1 mm diameter at extrusion rates of 5 mm s^−1^. The feed filament was extruded successfully through two different drive techniques: with a standard stepper motor driver or with applied air pressure of 300 kPa. Extrusion nozzle temperatures between 90 °C and 150 °C were used and the resulting extrusion forms were reprocessed several times to demonstrate recyclability. The material demonstrated highly tunable material properties and layer adhesion of extruded forms was observed, showing promise for future fused filament fabrication 3D printing applications. The custom extrusion print head was mounted to a KUKA Robotics Corporation 6 axis robotic arm (Fig. 1c) for basic 3D printing line tests of SRT and the same system at higher temperatures was used to successfully extrude more common fused filament fabrication materials such as acrylonitrile butadiene styrene, polylactic acid and nylon.

### Lattice organization at the molecular and nanoscopic levels

**Figure 2:**
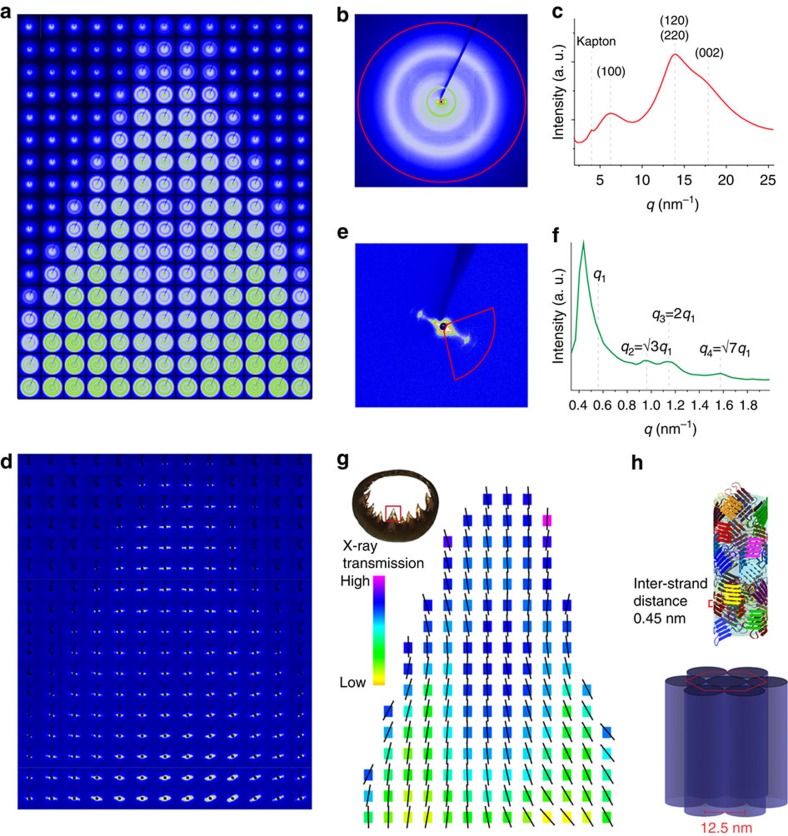
Multi-level X-ray scattering analysis of SRT structural features. (**a**) Mesh of multiple 2D WAXS patterns with steps of 100 μm covering the entire tip of a single sucker ring tooth. A representative 2D WAXS pattern (**b**) and the radial integration pattern of the intensity (**c**) as a function of the scattering vector *q*. The observed ring patterns confirm the random orientation of the β-sheet crystals. (**d**) Diffraction pattern mesh highlighting the SAXS region from **a**, clearly showing structural anisotropy at the nanoscale. (**e**,**f**) A representative 2D SAXS pattern (**e**) and the radial integration pattern of the intensity (**f**). The pattern reveals the fibrillar organization of the protein network and the positions of equatorial reflections suggest a hexagonal packing of the fibrils with an inter-fibrillar distance of 12.5 nm. (**g**) 2D colour-coded image depicting the mean fibrillar orientations (black lines) superimposed on an X-ray transmission data set. (**h**) Schematic model based on the X-ray scattering observations.

Molecular and nanoscale structural features of the native SRT were investigated using synchrotron X-ray scattering. Two-dimensional (2D) scattering pattern maps acquired from an individual sucker ring tooth at 100 μm lateral spatial resolution are displayed in Fig. 2a (wide-angle X-ray scattering (WAXS)) and Fig. 2d (small-angle X-ray scattering (SAXS)). A representative WAXS 2D pattern (Fig. 2b) exhibited diffuse rings, which is consistent with a random orientation of scattering domains, a theme maintained along the entire tooth. A quantitative analysis of the azimuthal integration of WAXS patterns in the *q*-range of 13–15 nm^−1^ (Supplementary Fig. 1) confirmed that the crystals were randomly oriented. The integrated intensity across the entire azimuth (*I* versus *q* plot, Fig. 2c) confirmed our previous results^21^, namely the presence of reflections at *q*=6.3, 13.9 and 17.9 nm^−1^ that can be attributed to nanoscale β-sheet domains embedded in an amorphous matrix. A closer inspection of the inter-strand distances provided values that were between those found in amyloid (0.465 nm) and silk-like β-sheet crystals (0.43 nm)^23^, suggesting a coexistence of these structural units in the SRT protein network.

To clarify the nanoscale organization of the protein network, the small angle regions of the X-ray scattering patterns were analysed in detail. SAXS mapping of the entire tooth (Fig. 2d) revealed that, in stark contrast to the isotropic orientation of β-sheets at the molecular scale, SRT exhibited strong anisotropy at the nanoscale, as exemplified by the ellipsoidal shapes of the 2D SAXS patterns (Fig. 2e), with the preferred orientation for each location represented with a line in Fig. 2g. A closer inspection of a representative SAXS pattern (Fig. 2e) clearly illustrated the anisotropy of the intensity profile, which could be related to the presence of elongated nanofibrils oriented roughly parallel to the tooth contour. Radial integration (Fig. 2f) of this pattern led to several reflections, which slightly varied in their scattering vector *q*, depending on the location, with values of *q*=0.94–0.97, 1.12–1.18 and 1.54–1.57 nm^−1^ that could be attributed to second-, third- and fourth-order Bragg reflections, while the first order was masked by the strong background at small *q*-values.

These reflections were consistent with an approximately hexagonal packing of elongated nanofibrils^24^, with small spatial variations. Based on the calculated average first-order *q*-value obtained from the experimental third-order reflection (1.16 nm^−1^) and the theoretical *q*-ratios of each reflection for a hexagonal packing^24^, we found an average *q*_1_ of 0.58 nm^−1^, corresponding to an inter-fibrillar distance of *ca*. 12.5 nm between adjacent fibrils. From the X-ray data, a plausible model for the molecular and nanoscale organization of the native SRT was created (Fig. 2h). Randomly oriented nanoconfined β-sheets are embedded in an amorphous protein network resulting in the isotropic structural stability of the nanofibrils. The nanofibrillar units are further assembled into a hexagonally packed lattice that runs predominantly parallel to the tooth contour, which may provide the high bending rigidity to the teeth required during prey capture and handling. This design strategy is not unique to the SRT and a similar architecture was previously reported for the mineralized nanofibrils of polychaete worm jaws, which are structurally organized to provide bending resistance^25^.

### Analysis of SRT thermo-mechanical properties

**Figure 3:**
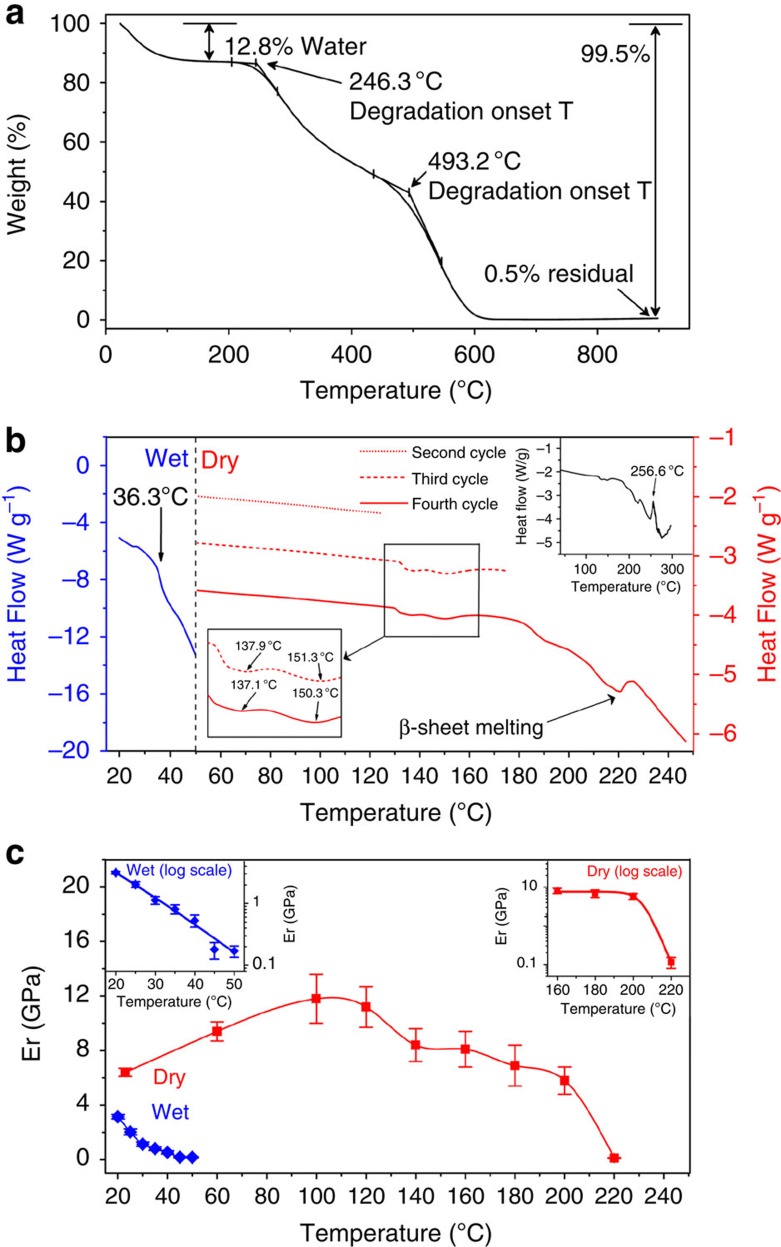
Thermo-mechanical properties of SRT. (**a**) Thermal gravimetric analysis (TGA) from RT up to 900 °C. Following water evaporation, SRT remain stable until *ca.* 250 °C. (**b**) DSC curves for hydrated (blue) and dry (red) samples. When hydrated, the heat flow was measured from RT to 60 °C. Under dry conditions, a first cycle (not shown) was ramped up to 120 °C, to remove residual water and subsequent cycles were ramped up to 120 °C (cycle 2), 170 °C (cycle 3) and 250 °C (cycle 4). Exothermic peaks at 137 °C and 151 °C were reproducible from cycle to cycle. The exothermic peak at 220 °C is attributed to β-sheet melting. (**c**) Reduced elastic modulus (*E*_r_) measured by high-temperature indentation using a temperature-ramping stage under both hydrated (blue) and dry (red) conditions.

Reliable processing of thermoplastics, including, for example, hot filament extrusion for 3D printing applications, requires a detailed understanding of a material's thermal behaviour. Thermal gravimetric analysis of the SRT revealed that they contained *ca*. 13 wt% water as illustrated by cumulative weight loss up to 100 °C (Fig. 3a). No additional significant weight loss occurred until *ca*. 250 °C, suggesting a broad range of SRT thermal stability. The results obtained from these measurements provided the suitable temperature range to subsequently investigate the thermo-mechanical response of the SRT. Using differential scanning calorimetry (DSC) and temperature-dependent nanoindentation measurements under both hydrated and dry conditions, we could link the mechanical response to the thermally induced phase transitions. Under hydrated conditions, a clear decrease in the slope of the DSC curve was detected at *ca*. 36 °C (Fig. 3b). In parallel, there was a clear reduction in elastic modulus from *ca*. 3 GPa to 200 MPa (Fig. 3c, inset) as the temperature rose from room temperature (RT) to 50 °C (which corresponded to the limit of stability of our nanoindenter for measurements under hydrated conditions). As β-sheets are stable up to *ca*. 220 °C as detailed below, the modulus decrease could be attributed to increased chain mobility of the amorphous domains in the presence of water.

In contrast, the thermo-mechanical behaviour of SRT differed significantly when the same measurements were performed under dry conditions. Here, the modulus increased with temperature up to 100 °C–110 °C, from 6 GPa to nearly 12 GPa, which is very high for a non-cross-linked thermoplastic polymer. As no phase transitions were detected by DSC up to 120 °C (Fig. 3b), we can unequivocally attribute this behaviour to residual water loss. As water is expelled from the structure, lattice compaction and loss of chain mobility ensues, thereby resulting in stiffening of the polymer network, a mechanism corroborated by the temperature-ramped X-ray scattering studies presented below. At higher temperature, DSC measurements revealed the presence of small exothermic peaks between 137 °C and 150 °C, which were fully reversible as illustrated by thermal cycling experiments during DSC (cycles 2 and 3), as well as an additional peak near 190 °C (Fig. 3b). Within this same temperature range, the modulus decreased only moderately and remained as high as 6 GPa at 200 °C (Fig. 3c), indicating minimal thermal damage. At 220 °C, however, another exothermal peak appeared on the DSC curve, which was correlated with a dramatic decrease in elastic modulus down to *ca*. 100 MPa. As discussed below from temperature-dependent WAXS and Fourier transform infrared (FTIR) spectroscopy measurements, this decrease can be attributed to the melting of β-sheets, in agreement with our previous measurements where chemical disruption of β-sheets also resulted in an order of magnitude reduction in modulus^21^. At temperatures higher than 220 °C, the SRT biopolymers lost their structural integrity and no successful indentation measurements could be performed.

### Temperature-dependent molecular and nanoscale changes

**Figure 4:**
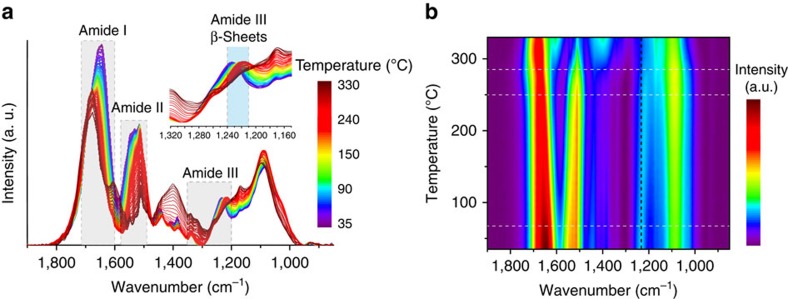
Tracking molecular interactions through *in situ* FTIR thermal treatment. (**a**) Representative FTIR spectra at temperatures ranging from 35 °C to 330 °C. These results provide insights into short-range molecular interactions and demonstrate the persistence of β-sheet structures (amide III) up to *ca.* 250 °C. Protein degradation occurs at *ca.* 300 °C. (**b**) Plot of the FTIR intensity as a function of energy (*x*) and temperature (*y*).

To further elucidate the molecular and nanoscale features associated with the thermoplastic behaviour of SRT, we conducted temperature-ramping FTIR, WAXS and SAXS measurements. FTIR spectra were collected from 35 °C to 330 °C and the temperature-dependent spectral changes are shown in Fig. 4a,b, represented as a 2D map of infrared intensity (wavenumber versus temperature). Analysis of the amide-specific bands in the FTIR spectra provides insight into secondary structure changes of protein networks^26^. Here we used the amide III band (1,200–1,350 cm^−1^) to investigate thermally activated transitions in the SRT proteins^27^,^28^. In these samples, the β-sheet-specific infrared band was centred at 1,235 cm^−1^ from RT up to *ca*. 150 °C (Fig. 4a, inset), at which point it shifted gradually to 1,220 cm^−1^, which was still within the β-sheet-specific region, suggesting only partial secondary structural rearrangement. Notably, all of these re-arrangements were fully reversible and the original peak positions were completely recovered after cooling the samples back to RT (Supplementary Fig. 2). Beyond 250 °C, major changes in all amide bands were observed. For example, the appearance of a band at 1,608 cm^−1^ in the amide I region and the disappearance of the amide III peak at 1,220 cm^−1^ (Fig. 4b, black dotted line) can be attributed to the formation of extended chain protein aggregates and to disruption of the β-sheet network, respectively^26^.

**Figure 5:**
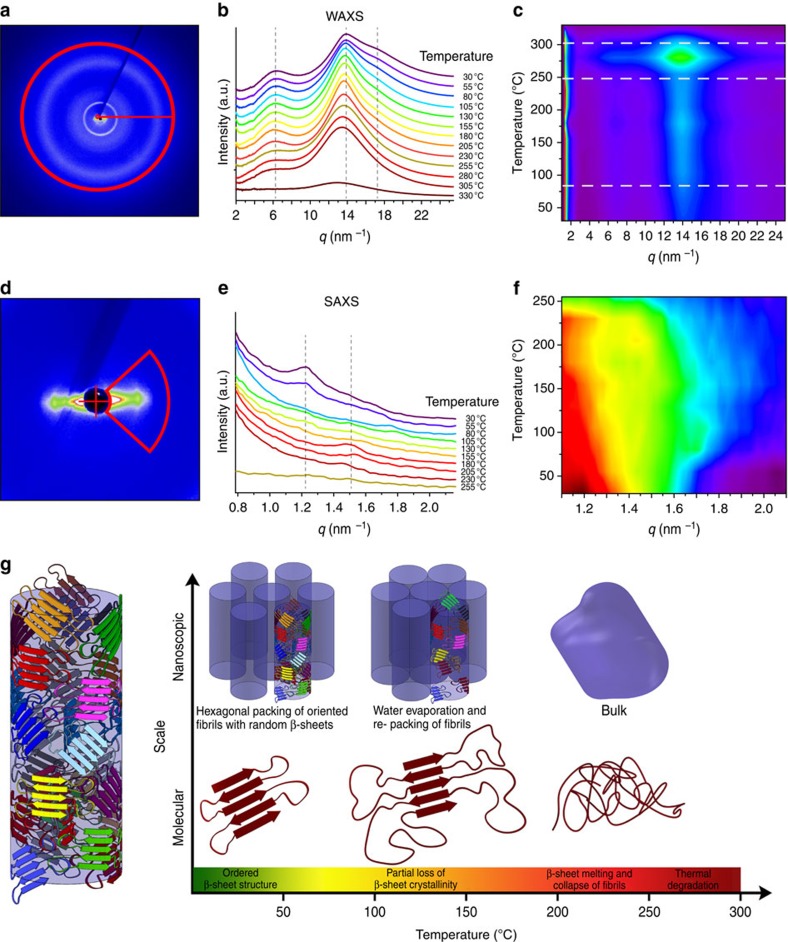
Tracking structural features through temperature-ramped *in situ* X-ray scattering. (**a**) WAXS pattern obtained from the tip of a single sucker ring tooth. (**b**) Scattering intensity plots calculated by radial integration (see red box in **a**) of WAXS patterns at various temperatures. Pronounced structural transformations are visible at *ca.* 80 °C (water removal), at *ca.* 230 °C (β-sheets melting) and at *ca.* 300 °C (polymer degradation). (**c**) Plot of radial integrated WAXS scattering intensity as a function of *q* (*x*) and temperature (*y*). (**d**) SAXS pattern obtained from the tip of a single sucker ring tooth. (**e**) Scattering intensity plots obtained by radial integration (see red box in **c**) of SAXS patterns at various temperatures. (**f**) Image of the radial integrated SAXS scattering intensity as a function of *q* (*x*) and temperature (*y*). Structural transformations at the level of β-sheets are visible at similar temperatures to those observed in the WAXS measurements. (**g**) Illustration of the thermally induced transformation of SRT at the molecular (bottom) and nanoscale (top) levels. All transformations are reversible until *ca.* 220 °C, at which point irreversible β-sheet melting and collapse of the nanofibrillar lattice occurs (see text for details).

Thermally induced structural transitions at the molecular scale were further probed by WAXS (Fig. 5a–c). Intensity plots (Fig. 5b) were obtained by radial integration of the 2D scattering patterns (Fig. 5a) and displayed as 2D intensity maps of scattering vector *q* versus temperature (Fig. 5c). At 80 °C, the 17.9 nm^−1^ reflection (002 plane) began to decrease in intensity and completely disappeared by 230 °C, whereas other reflections remained unaltered, suggesting slight disordering along the β-strand direction, as water was removed from the network. No other transitions were detected until 230 °C, where the main β-sheet reflection at *q*=13.9 nm^−1^ broadened slightly, indicating melting of the nanoconfined β-sheets. This transition was fully consistent with the DSC data and with the rapid degradation of mechanical properties at equivalent temperatures (Fig. 3c). The *q*=13.9 nm^−1^ reflection intensity further decreased between 250 °C and 300 °C (Fig. 5c), and the signal eventually disappeared by *T*>300 °C, in agreement with thermal gravimetric analysis measurements that indicated the onset of polymer thermal degradation near 250 °C. We note that the phase transitions detected by DSC at 138 °C and 151 °C leave the WAXS signal fundamentally unaffected, suggesting that these transformations are either associated with amorphous regions of the network or with rearrangement of the β-sheets at the nanoscale.

Structural changes at the level of fibrils were further assessed by analysing SAXS patterns as a function of temperature, as displayed in Fig. 5d–f. At 80 °C, for example, the 1.2 nm^−1^ reflection disappeared and no additional reflections were observed up to *ca*. 150 °C, indicating loss of the ordered hexagonal packing as water evaporated from the network. As the temperature further increased, new reflections appeared at higher *q-*values, suggesting structural rearrangement through compaction of the fibrillar lattice, and eventually the SAXS pattern completely disappeared at temperatures above 230 °C.

To assess whether the general network structure was maintained during processing, we drew high aspect ratio fibers (*ca*. 10 μm in diameter and a few centimeters long, see Methods) from SRT powder initially heated in water, and analysed their X-ray scattering profiles post processing. The resulting WAXS pattern was similar to that of the native pattern (Supplementary Fig. 3), indicating that the β-sheet structure was preserved, although the main reflections were slightly shifted towards higher *q*-values, suggesting small molecular re-arrangement of the β-sheets. Likewise, the post-drawing SAXS pattern was highly anisotropic (Supplementary Fig. 3), indicating that the general organization of the elongated nanofibrils was also persevered during drawing.

## Discussion

The ability of the SRT biopolymers to be moulded and extruded using traditional thermo-processing methods is intimately related to their primary protein structure and to their molecular and nanoscale thermal properties. In previous investigations^21^, we discovered that SRT are made of a novel family of proteins (called ‘suckerins'), exhibiting a distinct block co-polymer-like primary structure, with β-sheet forming sequences flanked by longer amorphous domains. The present study further refines this model across larger length scales and provides critical insight into the underlying principles of their thermoplastic behaviour. Each phase of the biphasic, semi-crystalline SRT biopolymer exhibits a specific thermal stability and affinity towards water or other potential plasticizing agents. At RT, the suckerins' molecular structure is similar to that of silks, where hydrophobic β-sheet nanocrystals that are likely to repel water are in turn surrounded by amorphous domains. Given the low molar concentration of hydrophilic residues in the amorphous region of the protein sequences, the total weight fraction of water remains modest at *ca*. 13 wt%. This water content, however, is critical for processability, and as inferred from thermo-mechanical measurements in the RT–120 °C range, water plays a key role as a plasticizing agent. Indeed, water evaporation from the network results in a very stiff material with an elastic modulus in the GPa range, which would be completely unsuitable for thermo-mechanical processing. As illustrated by our thermal extrusion studies, however, the optimal processing temperature of the SRT polymer can be increased dramatically through the use of plasticizers such as glycerol. Further optimization of plasticizer properties and evaporation/leeching techniques could thus ultimately result in more homogenous feed filament production for larger-scale/longer-term 3D printing-related applications.

At the nanoscale, the β-sheets are assembled within a nanofibrillar lattice. Although this lattice exhibits structural re-arrangement from *ca*. 100 °C to 150 °C, it remains thermally stable up to 220 °C, at which point β-sheet melting induces lattice collapse. As water is absent at high temperatures, these results imply that water plasticizes the amorphous domains without affecting the β-sheets, otherwise the stiffness reduction would be much more pronounced in the 120 °C–220 °C temperature range. By combining all of our temperature-dependant spectroscopic and scattering data, a model describing the thermal stability of SRT and its associated structural transitions can be proposed (Fig. 5g): the nanofibrillar structure made of semi-crystalline fibrils begins to re-arrange at 80 °C when water evaporates from the protein network. Water removal (including molecularly bound water) is complete by 150 °C and is accompanied by tighter nanofibrillar packing as observed in the SAXS patterns. At the molecular scale, transitions in this temperature range occur in the form of partial β-sheet rearrangement and disordering, possibly triggered by the increased mobility of the adjacent amorphous domains. The network remains stable until 220 °C, where the combined SAXS, WAXS and DSC data strongly suggest β-sheet melting within the nanofibrils, which in turn induces collapse of the nanofibrillar lattice. The observation that the SAXS pattern from drawn fibres was highly anisotropic, whereas at the same time the WAXS pattern associated with β-sheets was maintained (Supplementary Fig. 3) is consistent with a biphasic semi-crystalline polymer network, whereby heat-resistant nanocrystalline β-sheets embedded within an amorphous matrix exhibit viscous flow at temperatures below the melting or degradation point of the crystalline phase, with the flow resulting from chain re-arrangement in the amorphous regions. It is interesting to note that a class of supramolecular block co-polymers reminiscent of the natural design of suckerins, namely consisting of a stiff crystalline phase within a soft matrix, has recently been shown to exhibit excellent high-temperature processability^29^.

This study has key implications for the synthesis of bioinspired materials not only made from artificial SRT proteins^30^,^31^, but more generally for the *de novo* design of modular protein-based materials or block co-polymers with thermoplastic behaviour and re-processability. Synthetic biological production of SRT proteins (suckerins) thus holds exciting promise for additive manufacturing using bottom-up protein synthesis coupled with top-down spatial and material digital control. In addition, fully recyclable, eco-friendly substrates inspired from these studies could also be employed in printed wearable electronics or in other multi-purpose biomedical devices.

## Methods

### Sample collection and preparation

*D. gigas* were acquired from commercial sources (Baja California Sur, Mexico). SRT were removed manually and carefully rinsed in fresh water and stored at −20 °C prior to use.

### Differential scanning calorimetry

SRT protein powders were obtained by grinding sucker rings under liquid nitrogen and were then stored overnight at RT. The thermal behaviour of the SRT proteins was investigated with a differential scanning calorimeter (DSC Q10, TA Instruments Co.). Five milligrams of protein were subjected to four cycles of heating and cooling at a ramping rate of 10 °C min^−1^ under a nitrogen atmosphere: The first cycle involved ramping from RT to 125 °C and a return to RT; the second to fourth cycles involved ramping from RT to 125 °C, 175 °C and 225 °C, respectively, and returning to RT before the next cycle. Three-minute isothermal incubation at RT was employed between each ramp. In another trial, SRT proteins were heated from RT to 300 °C at a ramping rate of 10 °C min^−1^ after a pre-heating cycle from RT to 150 °C, to remove the residual water.

The glass transition temperature, Tg, of SRT proteins in water, was determined with the same instrument (DSC Q10). Fifty milligrams of SRT in distilled water (1:2 ratio w/w) were mixed and sealed in a 40-μl stainless steel pan. The temperature scanning range was from 10 °C to 90 °C at a heating rate of 5 °C min^−1^ under a nitrogen atmosphere.

### Thermo-gravimetric analysis

The decomposition profile of SRT proteins was characterized by thermogravimetric analysis (Q500, TA Instruments Co.). Twenty milligrams of SRT proteins were heated from RT to 900 °C at a ramping rate of 10 °C min^−1^ under an air atmosphere.

### Nanoindentation

The mechanical properties of SRT under dry and wet conditions at elevated temperatures were investigated by high-temperature nanoindenation. The measurements were performed with a TriboScan 950 (Hysitron) equipped with a thermal control system, which consisted of (a) a heating stage with a thermocouple positioned below the specimen, (b) a coolant system that was used to remove heat from the surrounding stage and (c) a temperature controller. SRT were embedded in a heat-conductive high-temperature chemical set cement (OMEGABOND OB-600) and polished with silicon carbide papers and polishing pastes (OP-S, Struers) down to 0.04 μm particle size. The resulting sectioned SRT samples exhibited a flat and smooth face, and a sample thickness of *ca*. 1 mm to reduce an intrinsic temperature gradient on heating. Under dry conditions, cross-sections of SRT were probed with a Berkovich high-temperature tip to a maximum load of 120 μN with loading and unloading rates of 24 μN s^−1^ and a holding time at peak load of 2 s using a piezo automation method. The temperatures were set at RT, 60 °C, 100 °C, 120 °C, 140 °C, 160 °C, 180 °C, 200 °C and 220 °C. Before the measurements at each elevated temperature were performed, the samples were preheated to each temperature for 30 min. Under wet conditions, the SRT cross-sections were probed with a cube corner elongated fluid cell tip to a maximum load of 80 μN with loading and unloading rates of 26.6 μN s^−1^ and a holding time at peak load of 2 s using a piezo automation method. The samples were first incubated in water for 1 h at RT, to achieve full hydration and measured before the temperature was increased to 50 °C at a rate of 5 °C per step. All modulus values were obtained using the classical Oliver-Pharr analysis method.

### Wide-angle and small-angle X-ray diffraction

WAXS and SAXS synchrotron radiation measurements were performed at the microfocus beamline at BESSY II (Helmholtz Zentrum, Berlin, Germany). X-ray scattering patterns were recorded with a 2D CCD (charge-coupled device) detector (MarMosaic 225, Rayonix, Inc., Evanston, USA) with a pixel size of 73 μm and an array of 3,072 × 3,072 pixels. The energy of the incident X-ray beam was 15 keV. The beam was further defined by a toroidal mirror together with a MoBC-multilayer monochromator with a pinhole size of 100 μm close to the sample^32^. For the sample mapping, a 60-μm step size was used in the scanning mode. The calibration (sample-to-detector distance, beam centre) and data evaluation were performed with DPDAK (DESY/MPIKG, Gunthard Benecke, v. 1.0.0) and OriginPro 8.6 was used for data plotting. WAXS and SAXS measurements were also conducted on fibres drawn from heated SRT powder. About 10 mg SRT were crushed into a powder using liquid N_2_, placed in 10 ml of deionized water and heated in a microwave oven resulting in a viscous melt. Fibres were then immediately drawn from the melt at a pulling rate of *ca*. 0.5 cm s^−1^, yielding micro-fibres about 1 cm long and 10 μm in diameter. About five fibres were bundled together and then glued between cardboard frames (1.5 cm × 1 cm) to secure them against damage during handling. Scattering data were acquired by focusing the X-ray beam onto the bundle.

### FTIR spectroscopy

For transmission FTIR spectra acquisition, the tip of one SRT was ground and the powder was mixed with dry pulverized KBr (Merck KGaA) and pressed into a pellet. The infrared spectra were acquired using a Vertex 70 FT-IR-Spectrometer (Bruker GmbH, Germany) with a Deuterated Triglycine Sulfate (DTGS) detector. A total of 32 scans were co-added per sample spectrum (wavenumber range: 600–4000, cm^−1^), background subtracted (concave rubberband correction, 2 iterations) and smoothed (9 smoothing points). For data processing and plotting, Opus 7.0 (Bruker) and OriginPro 8.6 were used.

### *In situ* thermal treatments

The *in situ* heating experiments were performed using a Linkam THMS600 heating stage. In the case of simultaneous X-ray scattering and thermal treatment, an intact sucker ring tooth was used. For FTIR transmission experiments, SRT powder was mixed with KBr and pressed into a pellet that was mounted on the heating stage. For the X-ray scattering experiments, the temperature was slowly raised to the desired values and diffractograms were acquired. For the FT-IR experiments, the temperature was automatically raised at 10 °C min^−1^ and an *in situ* time series of spectra were collected.

## Additional information

**How to cite this article:** Latza, V. *et al.* Multi-scale thermal stability of a hard thermoplastic protein-based material. *Nat. Commun.* 6:8313 doi: 10.1038/ncomms9313 (2015).

## Supplementary Material

Supplementary Information Supplementary Figures 1-3
