# Supplementary material for: Multi-scale thermal stability of a hard thermoplastic protein-based material
Source: Nat Commun. 2015 Sep 21;6:8313. doi: 10.1038/ncomms9313 (PMC4595719; doi:10.1038/ncomms9313)
Supplement: Supplementary Information — Supplementary Figures 1-3 [file ncomms9313-s1.pdf]

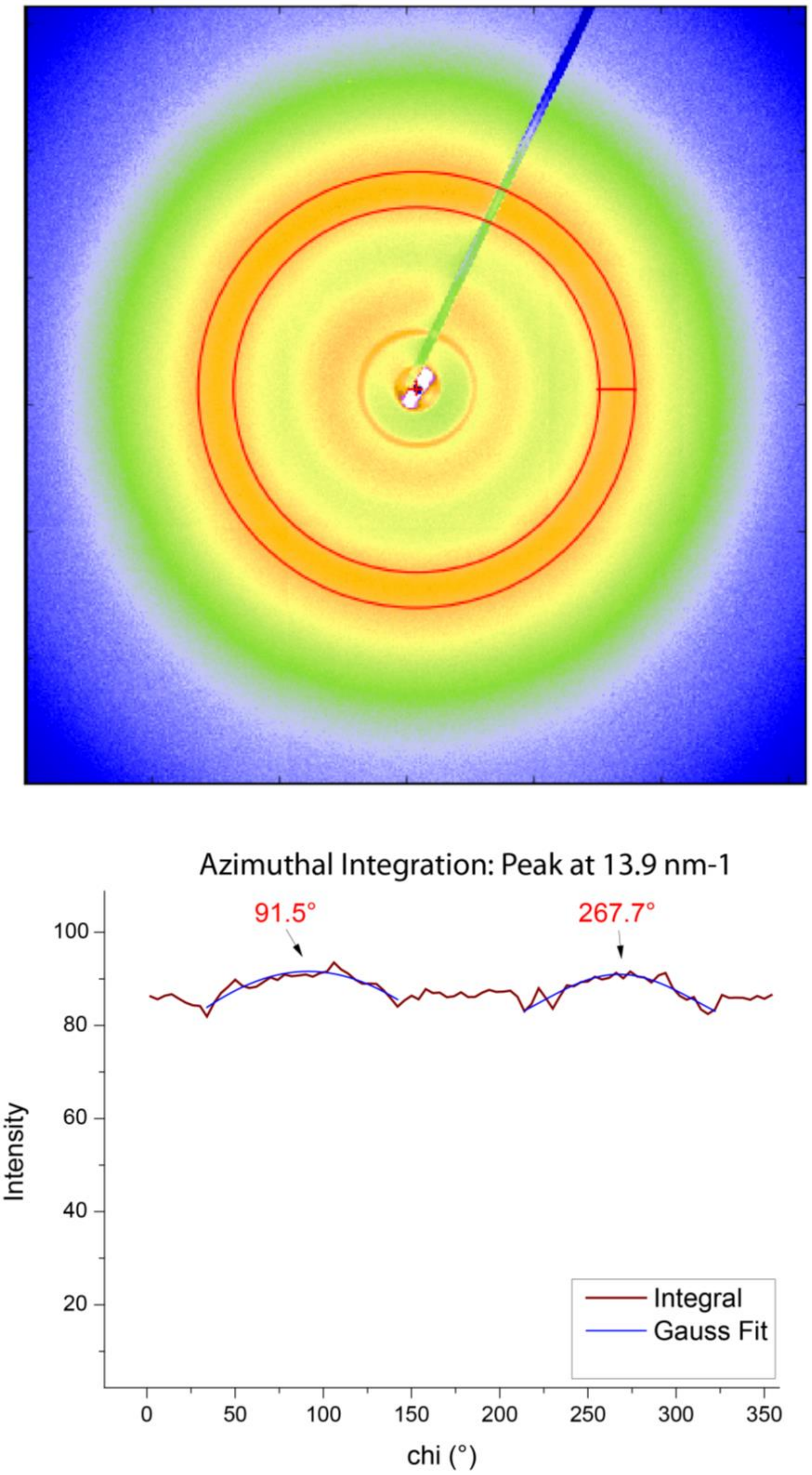

Supplementary Figure 1. Synchrotron-based wide-angle X-ray scattering (WAXS) of a native sucker ring tooth. Quantitative analysis of the azimuthal integration of the WAXS pattern in the *q*-range 13–15 nm^{-1}.

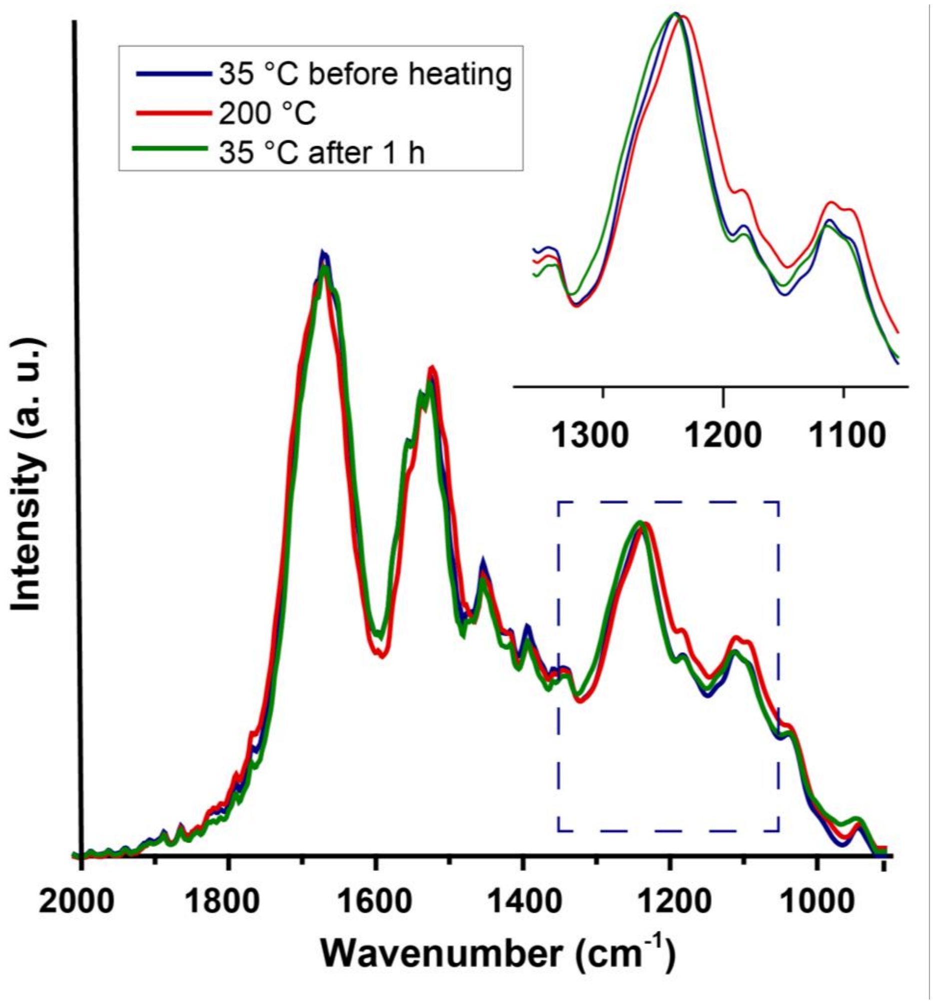

Supplementary Figure 2. Reversibility of the Fourier transform infrared (FTIR) spectral features of native sucker ring teeth (SRT) after heating to 200 °C followed by cooling for 1 h at 35 °C. Inset shows the magnified amide III spectral region.

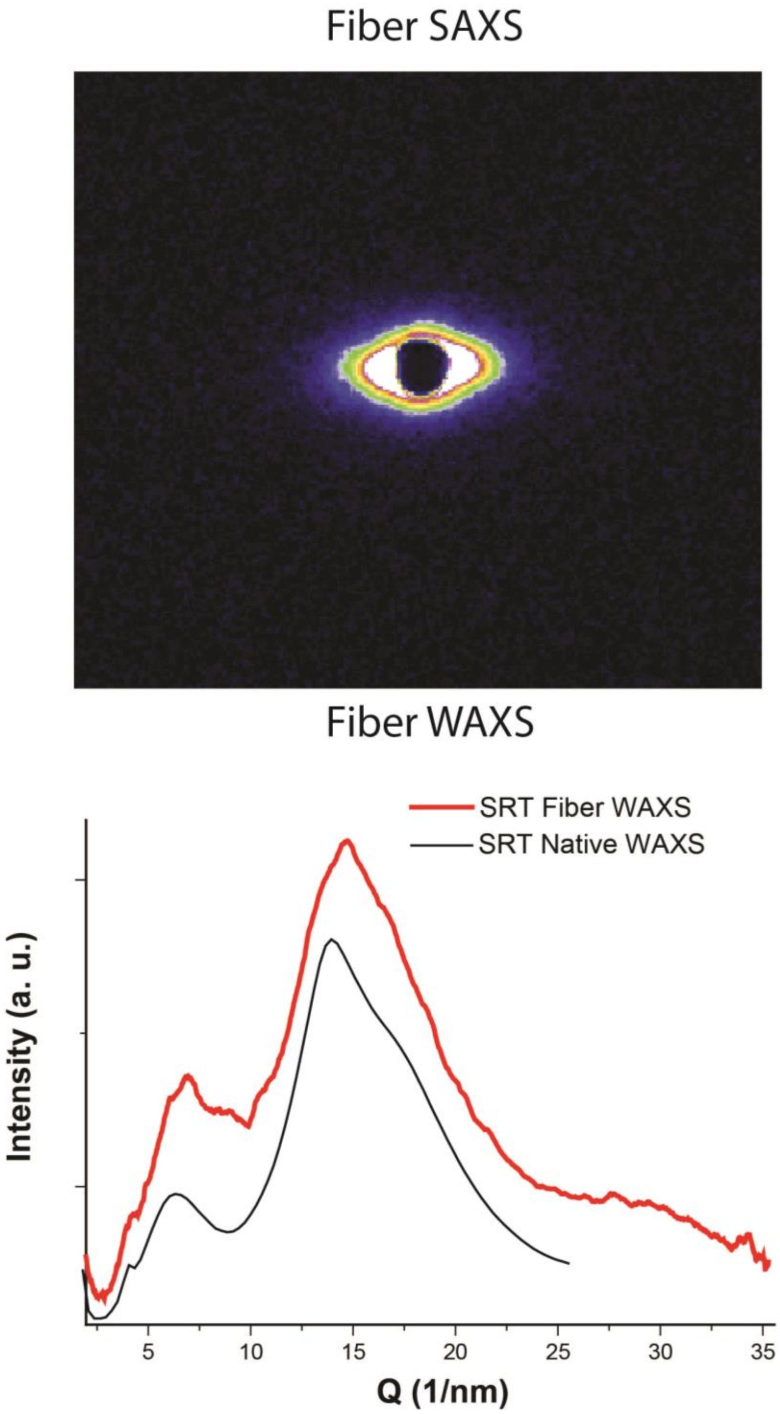

Supplementary Figure 3. Small-angle X-ray scattering (SAXS) pattern (top) and wide-angle X-ray scattering (WAXS) pattern (bottom, red line) of a micro-scale fiber drawn from SRT powder that was initially heated under hydrated conditions in a microwave for 30 seconds. The resulting WAXS (red line) pattern is compared with the WAXS pattern of the native SRT (black line).
